# Genomic sequence, organization and characteristics of a new nucleopolyhedrovirus isolated from *Clanis bilineata *larva

**DOI:** 10.1186/1471-2164-10-91

**Published:** 2009-02-25

**Authors:** Shan-Ying Zhu, Jian-Ping Yi, Wei-De Shen, Li-Qun Wang, Hua-Gang He, Yong Wang, Bing Li, Wen-Bing Wang

**Affiliations:** 1 Institute of Life Sciences, Jiangsu University, Zhenjiang 212013, PR China; 2 Shanghai Entry-Exit Inspection and Quarantine Bureau, Shanghai 200135, PR China; 3 School of Life Sciences, Soochow University, Suzhou 215123, PR China; 4 School of Food and Biological Engineering, Jiangsu University, Zhenjiang 212013, PR China

## Abstract

**Background:**

Baculoviruses are well known for their potential as biological agents for controlling agricultural and forest pests. They are also widely used as expression vectors in molecular cloning studies. The genome sequences of 48 baculoviruses are currently available in NCBI databases. As the number of sequenced viral genomes increases, it is important for the authors to present sufficiently detailed analyses and annotations to advance understanding of them. In this study, the complete genome of *Clanis bilineata *nucleopolyhedrovirus (ClbiNPV) has been sequenced and analyzed in order to understand this virus better.

**Results:**

The genome of ClbiNPV contains 135,454 base pairs (bp) with a G+C content of 37%, and 139 putative open reading frames (ORFs) of at least 150 nucleotides. One hundred and twenty-six of these ORFs have homologues with other baculovirus genes while the other 13 are unique to ClbiNPV. The 30 baculovirus core genes are all present in ClbiNPV. Phylogenetic analysis based on the combined *pif*-2 and *lef*-8 sequences places ClbiNPV in the Group II Alphabaculoviruses. This result is consistent with the absence of *gp*64 from the ClbiNPV genome and the presence instead of a fusion protein gene, characteristic of Group II. Blast searches revealed that ClbiNPV encodes a photolyase-like gene sequence, which has a 1-bp deletion when compared with photolyases of other baculoviruses. This deletion disrupts the sequence into two small photolyase ORFs, designated Clbi*phr*-1 and Clbi*phr*-2, which correspond to the CPD-DNA photolyase and FAD-binding domains of photolyases, respectively.

**Conclusion:**

ClbiNPV belongs to the Group II Alphabaculoviruses and is most closely related to OrleNPV, LdMNPV, TnSNPV, EcobNPV and ChchNPV. It contains a variant DNA photolyase gene, which only exists in ChchNPV, TnSNPV and SpltGV among the baculoviruses.

## Background

Baculoviruses are a large group of rod-shaped, enveloped viruses with circular, covalently closed, double-stranded DNA genomes. These viruses are pathogenic to arthropods, mainly insects within the orders Lepidoptera, Diptera and Hymenoptera [1,2]. According to morphology of the virus occlusion bodies (OBs), the family *Baculoviridae *comprises two genera: the Nucleopolyhedroviruses (NPVs) and Granuloviruses (GVs). The lepidopteron NPVs can be further divided into two sub-groups on the basis of their envelope fusion proteins, which are essential for the spread of infection in the insect and are required for efficient virus budding. Group I NPVs possess proteins related to GP64, whereas no GP64 homologues have been identified in Group II NPVs [3,4]. Instead, members of Group II encode homologues of LD130 proteins, also known as Fusion (F) proteins [5]. The taxonomy of the Baculoviridae genera has recently been changed on the basis of the hosts. There are now four genera: the Alphabaculoviruses (lepidopteron-specific NPV), Betabaculoviruses (lepidopteron-specific GV), Gammabaculoviruses (hymenopteron-specific NPV), and Deltabaculoviruses (dipteron-specific baculovirus) [6].

In recent years, much research has focused on baculoviruses owing to their potential as agents for biological control of pests in agriculture and forestry [7]. Furthermore, they can be used as efficient expression vectors of foreign genes [8,9]. Forty-eight completely-sequenced baculovirus genomes, including 34 Alphabaculoviruses, 10 Betabaculoviruses, 3 Gammabaculoviruses and 1 Deltabaculovirus (see Additional file 1), with sizes ranging from 81,755 base pairs (bp) in *Neodiprion lecontei *NPV (Nele NPV) [10] to 178,733 bp in *Xestia c-nigrum *GV (XecnGV) [11], have been made available in GenBank since the *Autographa californica *NPV (AcMNPV) genome sequence was reported [12].

*Clanis bilineata *(Walker), belonging to *Lepidoptera Sphingidae*, is a major agricultural pest causing considerable damage to soybean production in China. No baculovirus able to infect *C. bilineata *larvae was reported until 2006 [13], when a novel baculovirus named *Clanis bilineata *nucleopolyhedrovirus (ClbiNPV) was isolated and purified from the larvae of the sphingid *C. bilineata *infected with NPV. Transmission electron micrographs showed that this virus occludes single-enveloped nucleocapsids and hence is an SNPV [14]. The ClbiNPV genome comprises 135,454 bp and codes for 139 putative open reading frames (ORFs) with a minimum size of 150 nucleotides. In this report, we present the complete sequence and organization of the ClbiNPV genome and compare them to other baculoviruses through genomic and phylogenetic analyses.

## Results and discussion

### Nucleotide sequence analysis of the ClbiNPV genome

The genome of ClbiNPV has a size of 135,454 bp [GenBank: DQ504428], slightly smaller than that of *Spodoptera exigua *NPV (SeMNPV, 135,611 bp) [15]. ClbiNPV has a highly AT rich genome. Its overall G+C content is 37%, similar to that recorded for *Agrotis segetum *GV (AgseGV) and *Ecotropis obliqua *NPV (EcobNPV) [16], and higher only than those of *Adoxophyes honmai *NPV (AdhoNPV, 35%) [17] and *Adoxophyes orana *NPV (AdorNPV, 34%) among the Alphabaculovirus (see Additional file 1).

According to convention [18], the adenine residue at the translational ATG start codon of the polyhedrin gene (*polh*) was considered to be nucleotide number 1 of the genome, and successive nucleotides were numbered in the direction of the *polh *gene (see Additional file 2). Analysis of the ClbiNPV genome sequence led to the identification of 139 putative ORFs with 50 or more amino acids and minimal overlapping of adjacent ORFs. There are 60 ORFs with the same orientation as the polyhedrin gene, and 79 with the reverse orientation. Within 150 bp upstream of the ATG start codon, 34 ClbiNPV ORFs have baculovirus early promoter motifs (CAGT), 51 have late promoter motifs (TAAG), and 29 carry both these motifs.

**Figure 1:**
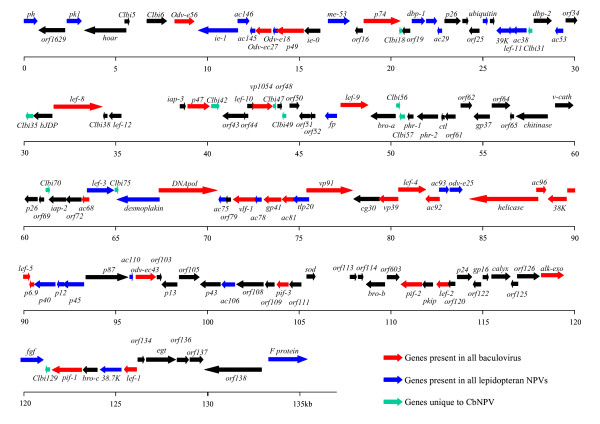
**Linear map of the 139 predicted ORFs for the complete ClbiNPV genome**. Arrows indicate ORFs and the direction of transcription. The names of putative genes are shown above or below the arrows. Numbers refer to the nucleotide position in kb (kilobases) relative to the start codon of the polyhedrin gene.

Of the 139 ClbiNPV ORFs identified, 126 are homologous to at least one other baculovirus, and the 30 core genes that are probably shared by all baculoviruses are conserved in the ClbiNPV genome [19]. Thirteen ORFs (Clbi5, Clbi6, Clbi18, Clbi31, Clbi35, Clbi42, Clbi47, Clbi49, Clbi56, Clbi57, Clbi70, Clbi75 and Clbi129) are unique to ClbiNPV; they account for 9% of the whole genome. Three baculovirus-repeated ORFs (*bro *genes) were identified in ClbiNPV (ORF55, 115 and 131) and were designated *bro-a*, *bro-b *and *bro-c*, respectively, based on their order in the genome. No typical homologous regions (hrs) were detected in ClbiNPV, which is similar to *Chrysodeixis chalcites *NPV (ChchNPV). The relative locations of these ORFs are shown diagrammatically in linear format in Figure 1. Their orientations, sizes and other details are shown in Additional file 2.

### Phylogenetic analyses and gene content

**Figure 2:**
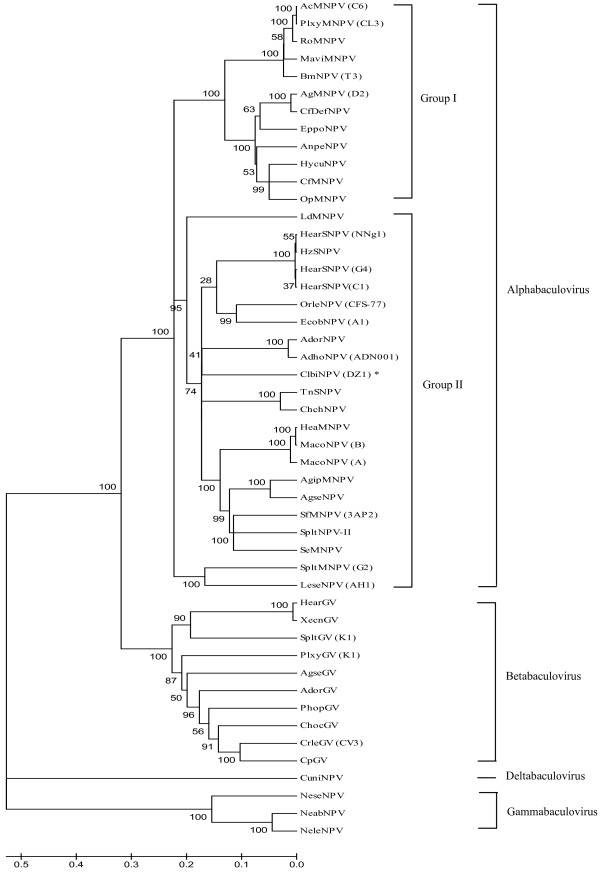
**Phylogenetic analysis using the predicted amino acid residues of 48 baculovirus PIF-2 and LEF-8 proteins**. An NJ (neighbor-joining) tree is shown. Numbers above or below the nodes are bootstrap values showing the statistical reliability of bootstrapping with 1,000 replicates.

The sequences of individual baculovirus genes such as polyhedrin/granulin (*polh*), *DNA polymerase*, *egt *and *gp41 *have previously been used for phylogenetic analysis [1]. Among those conserved baculovirus genes, *pif*-2 (*ac22 *in AcMNPV), encoding a *per os *infectivity factor, and *lef*-8, encoding a subunit of the baculovirus RNA polymerase, proved to be particularly reliable baculovirus markers for phylogenetic analyses at the virus family level [2]. A combined phylogenetic analysis of the *pif*-2 and *lef*-8 sequences further showed a clear, highly-supported classification among the four genera, and Alphabaculoviruses (lepidopteron-specific NPV) can be subdivided into Groups I and II. Phylogenetic analysis placed ClbiNPV in Group II (Figure 2). ORF 139 in the ClbiNPV genome encodes a typical F protein, and ClbiNPV does not encode GP64, which is consistent with its classification as a Group II Alphabaculovirus.

**Figure 3:**
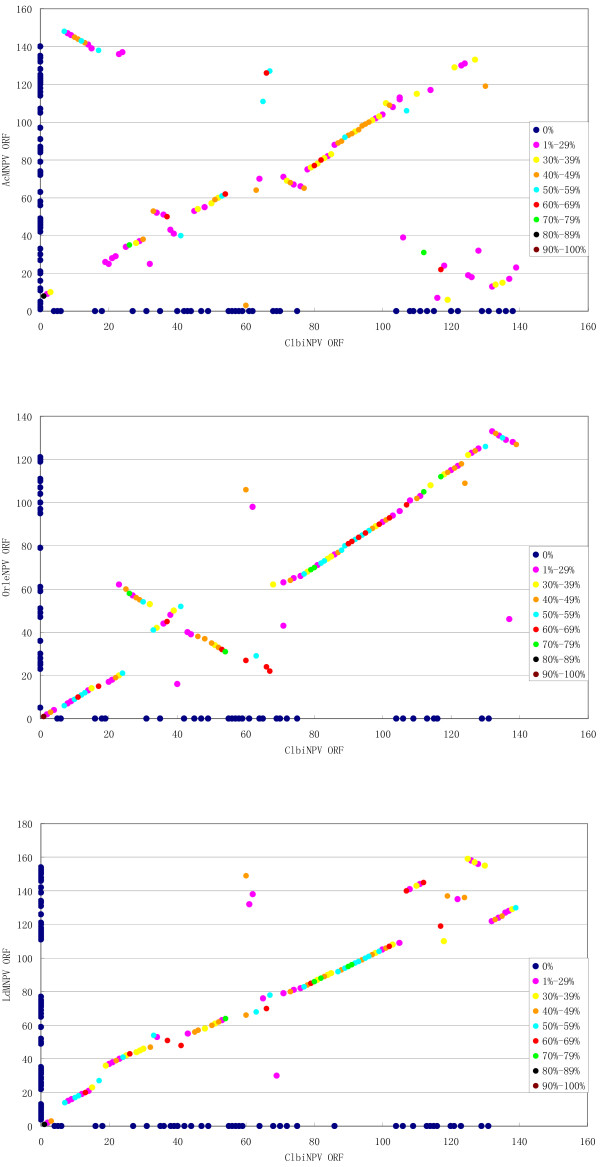
**Pairwise comparison of gene content and position of ClbiNPV with AcMNPV, OrleNPV and LdMNPV using gene parity plot analysis**. Genes present in only one of the two viruses in the pair-wise comparison appear on the X or Y axes.

The gene order of ClbiNPV was compared to AcMNPV [12], OrleNPV and LdMNPV [20] by gene parity plots [21]. ClbiNPV shared 102, 108 and 101 ORFs with AcMNPV, OrleNPV and LdMNPV, respectively. In general, gene order is conserved between ClbiNPV and LdMNPV, and between ClbiNPV and OrleNPV, although three inverted areas, involving Clbi25-32, Clbi43-60 and Clbi132-139, are identified when compared with OrleNPV. The genomes appear less collinear than AcMNPV (Figure 3).

### Overlapping ORF pairs in the sequenced Alphabaculovirusgenomes

Overlapping ORFs in ClbiNPV were searched and 26 pairs were found. The overlapping ORF pairs in all sequenced Alphabaculovirus genomes were further analysed (see Additional file 3).

In Group I Alphabaculoviruses, the numbers of overlapping ORF pairs range from 21 (BmNPV) to 42 (AgMNPV). Except for *ac68*/*ac69 *and *ac73*/*ac74 *in OpMNPV, and *ac43*/*ac44 *in HycuNPV, nine (*ac43*/*ac44*, *ac68*/*ac69*, *ac73*/*ac74*, *ac80*/*ac81*, *ac81*/*ac82*, *ac82*/*ac83*, *ac95*/*ac96*, *ac98*/*ac99 *and *ac102*/*ac103*) appear in all Group I Alphabaculoviruses.

In Group II Alphabaculoviruses, the numbers of overlapping ORF pairs range from 18 (EcobNPV and OrleNPV) to 42 (AgipMNPV). Except for several NPVs, six overlapping ORF pairs (*ac53a*/*ac54*, *ac57*/*ac59*, *ac67*/*ac68*, *ac80*/*ac81*, *ac82*/*ac83*, *ac89*/*ac90*) are conserved, and four pairs (*ac81*/*ac82*, *ac95*/*ac96*, *ac98*/*ac99 *and *ac102*/*ac103*) exist in all Group II Alphabaculoviruses.

Altogether, overlapping ORF pairs *ac81*/*ac82*, *ac95*/*ac96*, *ac98*/*ac99 *and *ac102*/*ac103 *were conserved in all Group I and Group II Alphabaculoviruses. These overlapping ORF pairs are from the coding regions of *ac81*, *tlp20*, *helicase*, *ac96*, *38K*, *lef*-5, *p12 *and *p45*, most of which have conserved functions in NPVs.

It was interesting that the numbers of overlapping ORFs in all Alphabaculoviruses were consistent with the phylogenetic tree constructed using the combined *pif*-2 and *lef*-8 sequences, meaning that closely-related NPVs have similar numbers of overlapping ORF pairs.

### DNA photolyase-like gene sequence with a 1-bp deletion

DNA photolyase is a monomeric protein that directly repairs lethal and carcinogenic UV-induced DNA lesions. It has been found in a variety of pathogens and other organisms [22]. However, among the baculoviruses, this enzyme exists only in ChchNPV [23,24], *Trichoplusia ni *NPV (TnSNPV) [25] and *Spodoptera litura *granulovirus (SpltGV).

**Figure 4:**
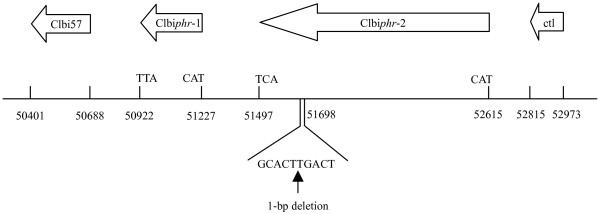
**ClbiNPV genome organization around the DNA photolyase locus**. ClbiNPV encodes a photolyase-like gene sequence, which has a 1-bp deletion when compared with photolyases of other baculoviruses. This deletion generates a premature termination codon at 51,497 and the next ATG begins at 51,227.

The complete ClbiNPV genome sequence analysis revealed the presence of a photolyase-like gene sequence 1,694 bp in length, which has the highest similarity to ChchNPV *phr*-2 using translated BLAST searches. Compared with photolyases among baculoviruses, it is interesting that there was a 1-bp deletion mutation (Figure 4). In order to confirm this deletion, we analyzed the DNA fragment library of ClbiNPV. There were seven colonies including the region with the mutation, and they all contained the 1-bp deletion. To ensure that this mutation was genuinely present in the original sample, PCR and sequencing of the gene region around this mutation were performed using the original ClbiNPV DNA. The sequencing results confirmed that the mutation occurred in this region. This 1-bp deletion mutation disrupted the sequence into two small ORFs, labelled *phr*-1 (Clbi58) and *phr*-2 (Clbi59) on the basis of their position in the ClbiNPV genome relative to the polyhedrin gene (Figure 4). The ORF of Clbi*phr*-1, corresponding to the 3' end of ChchNPV *phr*-2, is 306 bp long and encodes a polypeptide of 101 amino acids with a predicted molecular mass of 12.1 kDa. An early baculoviral transcription initiation motif (CAGT) was found 48 bp upstream of the putative translational start site (ATG), suggesting that Clbi*phr*-1 might be an early gene. In addition, a GATA motif (TGATAA) was found at position -129 bp relative to the translational start codon and might be involved in the transcriptional regulation of this gene. Clbi*phr*-2, corresponding to the 5' portion of ChchNPV *phr*-2, is 1,119 bp long and encodes a polypeptide of 372 amino acids with a predicted molecular mass of 42.0 kDa. No motifs characteristic of early (CAGT) or late (TAAG) baculovirus transcription initiation were found in the sequence upstream of Clbi*phr*-2. Two poly(A) motifs (AATAAA) are present at the end of the ORF of Clbi*phr*-2. Both the cyclobutane pyrimidine dimer (CPD)-DNA photolyase domain and the flavin adenine dinucleotide (FAD)-binding domain, characteristic of photolyases, are conserved in the ClbiNPV sequence. The CPD-DNA photolyase domain is located between amino acids 1 to 89 in Clbi*phr*-1, and an FAD-binding domain is located between amino acids 121 and 287 in Clbi*phr*-2.

Baculoviruses are attractive candidates for biological control of insect pests [26-28]. One major factor limiting their successful use in biological control is their sensitivity to inactivation by ultraviolet (UV) radiation [29]. The most significant cellular target of UV is DNA. When DNA is exposed to UV, it is damaged by producing pyrimidine dimers [30], which may block the activities of DNA or RNA polymerases and result in nucleotide misincorporation or inhibit polymerase progression during DNA replication or transcription [31-34]. DNA photolyase is a photo-reactivating enzyme that can repair the toxic effects of UV-induced DNA damage. van Oers et al. [23] suggested that the presence of a CPD-DNA photolyase gene in ChchNPV might be a remnant of the evolutionary history of baculoviruses, or a recent adaptation to a current ecological niche in *Chrysodeixis chalcites *or an alternative host, which might have given ChchNPV a competitive advantage. However, the functional significance of this gene in ChchNPV infection has not been proved. We analyzed the photolyase genes in baculoviruses and found that they are almost all early genes expressed before virus DNA replication. Therefore, we speculate that baculovirus photolyases might play a critical role in repairing DNA damage caused by UV, enabling the replication of virus DNA to complete successfully. In ClbiNPV genome, the photolyase-like gene sequence was split into two ORFs by the 1-bp deletion. However, at present, we cannot give direct evidence that this mutant affects the function, since no insect cell line permitting ClbiNPV infection has been found. Our further research will focus on confirming the expression pattern of the ClbiNPV photolyase gene and detecting photolyase activity in baculoviruses.

## Conclusion

Our preliminary studies on host range showed that ClbiNPV infects the larvae of *Clanis bilineata tiainglauica *Mell, *Ampelophaga rubiginosa *Bremer and Grey, *Theretra odenlandiae *(Fabricius) and *Pergesa elpenor lewisil*, and thus could be a candidate biological control agent for a broad spectrum of pests.

ClbiNPV is a Group II Alphabaculovirus and encodes 139 ORFs. Twenty-eight of these are best matched with the ORFs of OrleNPV, while 11 have the highest identities with LdMNPV, TnSNPV and AgseNPV. The numbers of ORFs best-matched with ChchNP, SfMNPV and EcobNPV are 8, 8 and 7, respectively. This coincides with the results of multi-alignment, phylogenetic analysis and the analyses of overlapping ORF pairs.

We also found a variant DNA photolyase-like gene sequence, which has a 1-bp deletion when compared with photolyases of other baculovirus. This deletion disrupted the sequence into two small photolyase ORFs, which correspond to the CPD-DNA and FAD-binding domains of photolyases, respectively. DNA photolyase may reduce lethal or mutagenic effects caused by ultraviolet radiation. This enzyme is present in the genomes of many species ranging from bacteria and yeasts to aplacental mammals such as the opossum [22,35]. In contrast, among the baculoviruses, it has been found only in ChchNPV, TnSNPV, SpltGV and ClbiNPV. Studies have shown that one major factor limiting the successful use of baculoviruses in biological control is their sensitivity to inactivation by UV radiation. The existence of DNA photolyase in these four baculovirus might have played an important role in their evolution and may reduce UV inactivation when applied in the field. However, further investigations are needed to understand the actual functional mechanism of this enzyme in baculoviruses.

## Methods

### Viruses

The occluded viruses were isolated from *C. bilineata *larva showing features typical of a nucleopolyhedrovirus infection in the field in Huzhou, Zhejiang Province.

### Purification of polyhedral inclusion bodies (PIBs)

The *C. bilineata *larva corpse was homogenized and diluted with sterilized double-distilled water. The dilution was filtered through three layers of cheesecloth to eliminate particulates. The filtrate was diluted with 50 mM Tris (pH 7.0) and centrifuged at 1,000 g for 5 min. The pellet was resuspended in Tris solution and again centrifuged at 1,000 g for 5 min. The dilution and centrifuging steps were repeated three times and the pellet was resuspended in Tris. SDS (sodium dodecyl sulfate) was added to the suspension to a final concentration of 0.2%, then incubated at room temperature for 30 min. Subsequently, the suspension was washed several times with Tris by following the above-mentioned steps. Finally, the pellet was resuspended in Tris solution.

### Preparation of nucleopolyhedrovirus DNA

The purified NPV PIBs were suspended in lysis buffer containing 0.1 M Na_2_CO_3_, 0.15 M NaCl and 0.01 M EDTA (pH 10.8) and incubated at room temperature for 30 min to dissolve the polyhedra. The pH of the suspension was adjusted to 8.0 with 10% acetic acid. Subsequently, SDS and proteinase K were added to final concentrations of 0.5% and 50 mg l^-1^, respectively, and incubated at 37°C overnight. The digested solution was extracted progressively with phenol, phenol and chloroform mixture, and chloroform, and DNA was precipitated with ethanol at a final concentration of 65%. The DNA was further purified and dissolved in 2.0 mM Tris (pH 8.0). The quantity and quality of the isolated DNA were determined spectrophotometrically and by electrophoresis on 0.7% agarose.

### DNA sequencing

A DNA fragment library of ClbiNPV was constructed through the shotgun method and the positive clones were sequenced by the Chinese National Human Genome Center at Shanghai. The purified viral DNA was sheared using an ultrasonic processor and blunt-ended using T4 DNA polymerase (TaKaRa). Fragments ranging from 1.6 to 4 kb were recovered from an agarose gel and ligated into the *Sma *I restriction site of pUC18. The ligation products were transformed into *Escherichia coli *DH10B by electroporation and the bacteria were grown on LB agar containing ampicillin, X-gal and IPTG. Recombinant colonies were picked randomly and DNA templates for sequencing were prepared using the 96-well plasmid preparation method. Both ends of the plasmid were sequenced using an ABI 3730 DNA Analyzer and six-fold coverage of viral DNA was obtained in this shotgun sequencing strategy.

### Sequence analysis

ORFs were identified using ORF finder . All BLAST searches were done through the National Center for Biotechnology Information (NCBI) websites. The phylogenetic tree for baculoviruses was based on the combined *pif*-2 and *lef*-8 sequences of the 48 baculoviruses, which were completely sequenced at the time of analysis, and the phylogenies were calculated using ClustalW alignments and MEGA4.0 (molecular evolutionary genetics analysis) software. Gene Parity Plot analysis was performed on the ClbiNPV genome versus the genomes of AcMNPV, LdMNPV and OrleNPV, as described previously [21,36].

## Authors' contributions

SYZ carried out the molecular cloning and analysis of these sequences and drafted the manuscript. JPY prepared the virus genomic library and designed the manuscript. WDS performed the statistical analysis and directed all the work of the manuscript. LQW prepared the virus genome and plasmids. HGH analyzed the data and drafted the manuscript. YW helped to draft the manuscript. BL participated in the sequence alignment. WBW carried out the design and draft of the manuscript. All authors read and approved the final manuscript.

## Supplementary Material

Additional file 1 **Characteristics of baculovirus genomes.** The data provided show the main characteristics of 48 completely-sequenced baculovirus genomes. Click here for file

Additional file 2 **ORFs predicted in the genome of ClbiNPV.** A detailed characteristics for all of the ORFs encoding putative proteins identified in ClbiNPV. Click here for file

Additional file 3 **Overlapping ORF pairs in the sequenced Alphabaculovirus genomes. **Overlapping ORFs in ClbiNPV were shown and the overlapping ORF pairs in all sequenced Alphabaculovirus genomes were analysed. Click here for file
