# Supplementary material for: Genomic sequence, organization and characteristics of a new nucleopolyhedrovirus isolated from Clanis bilineata larva
Source: BMC Genomics. 2009 Feb 25;10:91. doi: 10.1186/1471-2164-10-91 (PMC2650706; doi:10.1186/1471-2164-10-91)
Supplement: Additional file 1 — Characteristics of baculovirus genomes. The data provided show the main characteristics of 48 completely-sequenced baculovirus genomes. [file 1471-2164-10-91-S1.doc]

## Additional file 1 Characteristics of baculovirus genomes

| Group | Virus name | abbreviation | Length (bp) | Number of ORFs coded | GC content (%) | GenBank accession number |
| --- | --- | --- | --- | --- | --- | --- |
| Alphabaculovirus (Group I) | *Antheraea pernyi* NPV | AnpeNPV | 126,629 | 147 | 53 | DQ486030(2006) |
|  | *Anticarsia gemmatalis* NPV (D2) | AgMNPV (D2) | 132,239 | 152 | 44 | DQ813662(2006) |
|  | *Autographa californica* NPV (C6) | AcMNPV (C6) | 133,894 | 156 | 40 | L22858(1994) |
|  | *Bombyx mori* NPV (T3) | BmNPV (T3) | 128,413 | 143 | 40 | L33180(1996) |
|  | *Choristoneura fumiferana* DEF NPV | CfDefNPV | 131,160 | 149 | 45 | AY327402(2003) |
|  | *Choristoneura fumiferana* MNPV | CfMNPV | 129,593 | 146 | 50 | AF512031(2003) |
|  | *Epiphyas postvittana* NPV | EppoNPV | 118,584 | 136 | 40 | AY043265(2001) |
|  | *Hyphantria cunea* NPV | HycuNPV | 132,959 | 148 | 45 | AP009046(2006) |
|  | *Maruca vitrata* MNPV | MaviMNPV | 111,953 | 126 | 38 | EF125867(2006) |
|  | *Orgyia pseudotsugata* MNPV | OpMNPV | 131,995 | 152 | 55 | U75930(1997) |
|  | *Plutella xylostella* MNPV (CL3) | PlxyMNPV (CL3) | 134,417 | 152 | 40 | DQ457003(2006) |
|  | *Rachiplusia ou* MNPV | RoMNPV | 131,526 | 149 | 39 | AY145471(2002) |
| Alphabaculovirus (Group II) | *Adoxophyes honmai* NPV (ADN001) | AdhoNPV (ADN001) | 113,220 | 125 | 35 | AP006270(2003) |
|  | *Adoxophyes orana* NPV | AdorNPV | 111,724 | 121 | 34 | EU591746(2008) |
|  | *Agrotis ipsilon* MNPV | AgipMNPV | 155,122 | 163 | 48 | EU839994(2008) |
|  | *Agrotis segetum* NPV | AgseNPV | 147,544 | 153 | 45 | DQ123841(2006) |
|  | *Chrysodeixis chalcites* NPV | ChchNPV | 149,622 | 151 | 39 | AY864330(2005) |
|  | *Clanis bilineata* NPV (DZ1) | ClbiNPV (DZ1) | 135,454 | 139 | 37 | DQ504428(2006) |
|  | *Ecotropis obliqua* NPV (A1) | EcobNPV (A1) | 131,204 | 126 | 37 | DQ837165(2006) |
|  | *Helicoverpa armigera* MNPV | HearMNPV | 154,196 | 162 | 40 | EU730893(2008) |
|  | *Helicoverpa armigera* NPV (C1) | HearSNPV (C1) | 130,759 | 137 | 38 | AF303045(2001) |
|  | *Helicoverpa armigera* NPV (G4) | HearSNPV (G4) | 131,405 | 135 | 39 | AF271059(2001) |
|  | *Helicoverpa armigera* SNPV (NNg1) | HearSNPV (NNg1) | 132,425 | 143 | 39 | AP010907(2008) |
|  | *Helicoverpa zea* SNPV | HzSNPV | 130,869 | 139 | 39 | AF334030(2002) |
|  | *Leucania separata* NPV (AH1) | LeseNPV (AH1) | 168,041 | 169 | 48 | AY394490(2006) |
|  | *Lymantria dispar* NPV | LdMNPV | 161,046 | 164 | 57 | AF081810(1998) |
|  | *Mamestra configurata* NPV (A) | MacoNPV (A) | 155,060 | 169 | 41 | U59461(1997) |
|  | *Mamestra configurata* NPV (B) | MacoNPV (B) | 158,482 | 168 | 40 | AY126275(2002) |
|  | *Orgyia leucostigma* NPV (CSF-77) | OrleNPV (CSF-77) | 156,179 | 135 | 39 | EU309041(2008) |
|  | *Spodoptera exigua* NPV | SeMNPV | 135,611 | 139 | 43 | AF169823(1999) |
|  | *Spodoptera frugiperda* MNPV (3AP2) | SfMNPV (3AP2) | 131,330 | 142 | 40 | EF035042(2007) |
|  | *Spodoptera litura* NPV (G2) | SpltMNPV (G2) | 139,342 | 141 | 42 | AF325155(2001) |
|  | *Spodoptera litura* NPV II | SpltNPV II | 148,634 | 147 | 44 | EU780426(2008) |
|  | *Trichoplusia ni* SNPV | TnSNPV | 134,394 | 145 | 38 | DQ017380(2005) |
| Betabaculovirus | *Adoxophyes orana* GV | AdorGV | 99,657 | 119 | 34 | AF547984(2003) |
|  | *Agrotis segetum* GV | AgseGV | 131,680 | 132 | 37 | AY522332(2004) |
|  | *Choristoneura occidentalis* GV | ChocGV | 104,710 | 116 | 32 | DQ333351(2006) |
|  | *Cryptophlebia leucotreta* GV (CV3) | CrleGV (CV3) | 110,907 | 128 | 32 | AY229987(2003) |
|  | *Cydia pomonella* GV | CpGV | 123,500 | 143 | 45 | U53466(2001) |
|  | *Helicoverpa armigera* GV | HearGV | 169,794 | 179 | 40 | EU255577(2008) |
|  | *Phthorimaea operculella* GV | PhopGV | 119,217 | 130 | 35 | AF499596(2002) |
|  | *Plutella xylostella* GV (K1) | PlxyGV (K1) | 100,999 | 120 | 40 | AF270937(2000) |
|  | *Spodoptera litura* GV (K1) | SpltGV (K1) | 124,121 | 136 | 38 | DQ288858(2007) |
|  | *Xestia c-nigrum* GV | XecnGV | 178,733 | 181 | 40 | AF162221(2000) |
| Gammabaculovirus | *Neodiprion abietis* NPV | NeabNPV | 84,264 | 93 | 33 | DQ317692(2006) |
|  | *Neodiprion sertifer* NPV | NeseNPV | 86,462 | 90 | 33 | AY430810(2004) |
|  | *Neodiprion lecontei* NPV | NeleNPV | 81,755 | 89 | 33 | AY349019(2004) |
| Deltabaculovirus | *Culex nigripalpus* NPV | CuniNPV | 108,252 | 109 | 50 | AF403738(2001) |
