# Supplementary material for: Genomic sequence, organization and characteristics of a new nucleopolyhedrovirus isolated from Clanis bilineata larva
Source: BMC Genomics. 2009 Feb 25;10:91. doi: 10.1186/1471-2164-10-91 (PMC2650706; doi:10.1186/1471-2164-10-91)
Supplement: Additional file 2 — ORFs predicted in the genome of ClbiNPV. A detailed characteristics for all of the ORFs encoding putative proteins identified in ClbiNPV. [file 1471-2164-10-91-S2.doc]

## Additional file 2 ORFs predicted in the genome of ClbiNPV

| ORF | Location | Length (aa) | *Name* | Prom.E/L | Homologous ORF number / amino acid identity (%) | | | | | | | | Best matched baculovirus ORF |
| --- | --- | --- | --- | --- | --- | --- | --- | --- | --- | --- | --- | --- | --- |
|  |  |  |  |  | AcMNPV | |  | LdMNPV | |  | OrleNPV | |  |
|  |  |  |  |  | ORF | ID(%) |  | ORF | ID(%) |  | ORF | ID(%) |  |
| 1 | 1→741 | 246 | *polyhedrin* | L | 8 | 85 |  | 1 | 80 |  | 1 | 90 | Busu ph |
| 2 | 738←2177 | 479 | *orf1629* | ? | 9 | 16 |  | 2 | 16 |  | 2 | 19 | Orle2 |
| 3 | 2235→3029 | 264 | *pk1* | E,L | 10 | 37 |  | 3 | 45 |  | 3 | 46 | Agse3 |
| 4 | 3253←5526 | 757 | *hoar* | E | － | － |  | － | － |  | 4 | 16 | Ecob4 |
| 5 | 5450→5710 | 86 | ***Clbi5*** | E | － | － |  | － | － |  | － | － | － |
| 6 | 6705→7784 | 359 | ***Clbi6*** | L | － | － |  | － | － |  | － | － | － |
| 7 | 8197→9261 | 354 | *odv-e56* | L | 148 | 53 |  | 14 | 53 |  | 6 | 55 | Ag144 |
| 8 | 9460←11628 | 722 | *ie-1* | E | 147 | 24 |  | 15 | 24 |  | 7 | 27 | Ecob6 |
| 9 | 11700→12326 | 208 | *ac146* | L | 146 | 23 |  | 16 | 26 |  | 8 | 26 | Se133 |
| 10 | 12347←12625 | 92 | *ac145* | L | 145 | 42 |  | 17 | 52 |  | 9 | 55 | Sf138 |
| 11 | 12709←13554 | 281 | *odv-ec27* | E,L | 144 | 45 |  | 18 | 59 |  | 10 | 62 | Orle10 |
| 12 | 13698←13967 | 89 | *odv-e18* | L | 143 | 52 |  | 19 | 23 |  | 11 | 57 | Ld19 |
| 13 | 13912←15408 | 498 | *p49* | L | 142 | 47 |  | 20 | 61 |  | 12 | 52 | Ld20 |
| 14 | 15478←16323 | 281 | *ie-0* | L | 141a | 24 |  | 21 | 24 |  | 13 | 23 | Sf143 |
| 15 | 16787→17956 | 389 | *me-53* | E,L | 139 | 14 |  | 23 | 33 |  | 14 | 37 | Orle14 |
| 16 | 18102←18503 | 133 |  | ? | － | － |  | 26 | 29 |  | － | － | Agse145 |
| 17 | 18543→20522 | 659 | *p74* | E | 138 | 55 |  | 27 | 56 |  | 15 | 60 | Adho27 |
| 18 | 20479←20640 | 53 | ***Clbi18*** | ? | － | － |  | － | － |  | － | － | － |
| 19 | 20646←21056 | 136 |  | E,L | 26 | 24 |  | 36 | 31 |  | － | － | Sf126 |
| 20 | 21191→21889 | 232 | *dbp-1* | E | 25 | 20 |  | 37 | 19 |  | 17 | 25 | Adho28 |
| 21 | 21904→22485 | 193 | *lef-6* | L | 28 | 20 |  | 38 | 26 |  | 18 | 24 | MacoA156 |
| 22 | 22558←22800 | 80 | *ac29* | L | 29 | 28 |  | 39 | 49 |  | 19 | 40 | Agse141 |
| 23 | 22966→23829 | 287 | *p26* | L | 136 | 28 |  | 40 | 25 |  | 20/62 | 38/18 | MacoA158 |
| 24 | 23925→24236 | 103 | *p10* | L | 137 | 18 |  | 41 | 55 |  | 21 | 53 | Adho32 |
| 25 | 24315←24866 | 183 |  | L | 34 | 22 |  | 42 | 36 |  | 60 | 46 | Adho18 |
| 26 | 24999→25250 | 83 | *ubiquitin* | L | 35 | 74 |  | 43 | 69 |  | 58 | 74 | Se123 |
| 27 | 25306→25539 | 77 |  | L | － | － |  | － | － |  | 57 | 26 | Adho15 |
| 28 | 25625←26470 | 281 | *39K/pp31* | E | 36 | 30 |  | 44 | 37 |  | 56 | 40 | Orle56 |
| 29 | 26406←26816 | 136 | *lef-11* | E,L | 37 | 21 |  | 45 | 39 |  | 55 | 41 | Agse131 |
| 30 | 26741←27469 | 242 | *ac38* | E,L | 38 | 44 |  | 46 | 47 |  | 54 | 52 | MacoB146 |
| 31 | 27544←27741 | 65 | ***Clbi31*** | ? | － | － |  | － | － |  | － | － | － |
| 32 | 27838→28794 | 318 | *dbp-2* | E,L | 25 | 24 |  | 47 | 42 |  | 53 | 33 | Ld47 |
| 33 | 28984←29406 | 140 | *ac53* | L | 53 | 43 |  | 54 | 52 |  | 41 | 58 | Orle41 |
| 34 | 29451→30056 | 201 |  | ? | 52 | 15 |  | 53 | 22 |  | 42 | 30 | Orle42 |
| 35 | 30053←30427 | 124 | ***Clbi35*** | E | － | － |  | － | － |  | － | － | － |
| 36 | 30476←31498 | 340 | *bJDP* | E | 51 | 17 |  | － | － |  | 44 | 21 | Orle44 |
| 37 | 31617→34256 | 879 | *lef-8* | E | 50 | 61 |  | 51 | 63 |  | 45 | 68 | Orle45 |
| 38 | 34392←34604 | 70 |  | E,L | 43 | 25 |  | － | － |  | 48 | 28 | Sf114 |
| 39 | 34725←35384 | 219 | *lef-12* | L | 41 | 23 |  | － | － |  | 50 | 31 | Orle50 |
| 40 | 38486→38785 | 99 | *iap-3* | E,L | － | － |  | － | － |  | 16 | 12 | CrleGV16 |
| 41 | 38982→40169 | 395 | *p47* | E | 40 | 51 |  | 48 | 60 |  | 52 | 56 | Sf116 |
| 42 | 40275→40700 | 141 | ***Clbi42*** | E | － | － |  | － | － |  | － | － | － |
| 43 | 40840←42006 | 388 |  | E,L | － | － |  | 55 | 20 |  | 40 | 26 | Chch42 |
| 44 | 42003←42275 | 90 |  | E,L | － | － |  | － | － |  | 39 | 29 | Agse115 |
| 45 | 42223→42591 | 122 | *lef-10* | E | 53a | 26 |  | 56 | 40 |  | － | － | Chch44 |
| 46 | 42455→43531 | 358 | *vp1054* | E | 54 | 33 |  | 57 | 45 |  | 38 | 41 | Ecob39 |
| 47 | 43616→43765 | 49 | ***Clbi47*** | E | － | － |  | － | － |  | － | － | － |
| 48 | 43804→44037 | 77 |  | E | 55 | 29 |  | 58 | 31 |  | 37 | 40 | HearG4 48 |
| 49 | 43998←44198 | 66 | ***Clbi49*** | ? | － | － |  | － | － |  | － | － | － |
| 50 | 44556→45077 | 173 |  | ? | 57 | 36 |  | 60 | 46 |  | 35 | 41 | Ld60 |
| 51 | 45055←45636 | 193 |  | E,L | 59 | 48 |  | 61 | 37 |  | 34 | 33 | Chch49 |
| 52 | 45726←45989 | 87 |  | ? | 60 | 33 |  | 62 | 42 |  | 33 | 43 | HearG4 52 |
| 53 | 46468←47094 | 208 | *fp/25K* | E,L | 61 | 52 |  | 63 | 26/20 |  | 32 | 63 | Se98 |
| 54 | 47253→48737 | 494 | *lef-9* | ? | 62 | 63 |  | 64 | 70 |  | 31 | 73 | Tn49 |
| 55 | 48851←50200 | 449 | *bro-a* | ? |  |  |  |  |  |  |  |  | HearGV54 |
| 56 | 50210→50404 | 64 | ***Clbi56*** | L | － | － |  | － | － |  | － | － | － |
| 57 | 50401←50688 | 95 | ***Clbi57*** | E | － | － |  | － | － |  | － | － | － |
| 58 | 50922←51227 | 101 | *phr-1* | ? | － | － |  | － | － |  | － | － | Chch72 |
| 59 | 51497←52615 | 372 | *phr-2* | E | － | － |  | － | － |  | － | － | Chch72 |
| 60 | 52815←52973 | 52 | *ctl* | E,L | 3 | 42 |  | 66/149 | 43/40 |  | 27/106 | 68/42 | Orps30 |
| 61 | 53016←53573 | 185 |  | E | － | － |  | 132 | 16 |  | － | － | Ld132 |
| 62 | 53802→54401 | 199 |  | E | － | － |  | 138 | 20 |  | 98 | 24 | CypoGV16 |
| 63 | 54469←55329 | 286 | *gp37* | L | 64 | 46 |  | 68 | 52 |  | 29 | 56 | MacoA37 |
| 64 | 55427→56416 | 329 |  | L | 70 | 14 |  | － | － |  | － | － | Ro68 |
| 65 | 56489←56692 | 67 |  | ? | 111 | 51 |  | 76 | 19 |  | － | － | Anpe46 |
| 66 | 56916←58622 | 568 | *chitinase* | L | 126 | 64 |  | 70 | 64 |  | 24 | 63 | Orle24 |
| 67 | 58919→59896 | 325 | *v-cath* | E,L | 127 | 57 |  | 78 | 56 |  | 22 | 62 | Orle22 |
| 68 | 60024←60713 | 229 | *p26* | ? | － | － |  | － | － |  | 62 | 36 | Orle62 |
| 69 | 60814←61080 | 88 |  | L | 150 | 21 |  | 30 | 20 |  | － | － | XecnGV20 |
| 70 | 61115→61321 | 68 | ***Clbi70*** | E | － | － |  | － | － |  | － | － | － |
| 71 | 61255←62175 | 306 | *iap-2* | E | 71 | 20 |  | 79 | 29 |  | 43/63 | 15/27 | Orle63 |
| 72 | 62182←63024 | 280 |  | ? | 69 | 39 |  | － | － |  | － | － | Se89 |
| 73 | 63103←63483 | 126 | *ac68* | E | 68 | 41 |  | 80 | 43 |  | 64 | 47 | Agse97 |
| 74 | 63347→64795 | 482 | *lef-3* | L | 67 | 21 |  | 81 | 25 |  | 65 | 29 | Sf90 |
| 75 | 64886→65065 | 59 | ***Clbi75*** | ? | － | － |  | － | － |  | － | － | － |
| 76 | 65007←67322 | 771 | *desmoplakin* | L | 66 | 15 |  | 82 | 23 |  | 66 | 19 | Ld82 |
| 77 | 67327→70509 | 1060 | *DNApol* | E | 65 | 43 |  | 83 | 54 |  | 67 | 52 | Agse100 |
| 78 | 70768←71160 | 130 | *ac75* | L | 75 | 28 |  | 84 | 43 |  | 68 | 32 | MacoA116 |
| 79 | 71169←71426 | 85 |  | L | 76 | 36 |  | 85 | 62 |  | 69 | 73 | Orle69 |
| 80 | 71541←72749 | 402 | *vlf-1* | L | 77 | 68 |  | 86 | 70 |  | 70 | 73 | Orle70 |
| 81 | 72742←73086 | 114 | *ac78* | L | 78 | 32 |  | 87 | 34 |  | 71 | 24 | HearG4 72 |
| 82 | 73190←74122 | 310 | *gp41* | L | 80 | 61 |  | 88 | 73 |  | 72 | 57 | Ld88 |
| 83 | 74125←75078 | 317 | *ac81* | L | 81 | 38 |  | 89 | 48 |  | 73 | 53 | Tn74 |
| 84 | 74711←75580 | 289 | *tlp20/ac82* | ? | 82 | 23 |  | 90 | 35 |  | 74 | 31 | Tn75 |
| 85 | 75432→77933 | 833 | *vp91* | L | 83 | 34 |  | 91 | 36 |  | 75 | 37 | Tn76 |
| 86 | 78077←79117 | 346 | *cg30* | ? | 88 | 19 |  | － | － |  | 76 | 27 | Orle76 |
| 87 | 79278←80330 | 350 | *vp39* | L | 89 | 40 |  | 92 | 50 |  | 77 | 47 | Ld92 |
| 88 | 80332→81825 | 497 | *lef-4* | E | 90 | 42 |  | 93 | 45 |  | 78 | 50 | Orle78 |
| 89 | 81942←82700 | 252 | *ac92* | ? | 92 | 51 |  | 94 | 55 |  | 80 | 54 | Ecob71 |
| 90 | 82699→83214 | 171 | *ac93* | L | 93 | 41 |  | 95 | 72 |  | 81 | 63 | Ld95 |
| 91 | 83216→83896 | 226 | *odv-e25* | E,L | 94 | 40 |  | 96 | 70 |  | 82 | 64 | Chch86 |
| 92 | 84289←88014 | 1241 | *helicase* | E,L | 95 | 38 |  | 97 | 54 |  | 83 | 58 | Orle83 |
| 93 | 87983→88501 | 172 | *ac96* | ? | 96 | 43 |  | 98 | 56 |  | 84 | 63 | Orle84 |
| 94 | 88625←89635 | 336 | *38K* | L | 98 | 40 |  | 99 | 49 |  | 85 | 54 | Chch91 |
| 95 | 89609→90355 | 248 | *lef-5* | ? | 99 | 48 |  | 100 | 52 |  | 86 | 62 | Ecob78 |
| 96 | 90349←90591 | 80 | *p6.9* | L | 100 | 44 |  | 101 | 53 |  | 87 | 53 |  |
| 97 | 90636←91766 | 376 | *p40* | E,L | 101 | 35 |  | 102 | 48 |  | 88 | 44 | Ld102 |
| 98 | 91914←92276 | 120 | *p12* | E,L | 102 | 22 |  | 103 | 36 |  | 89 | 32 | Ecob81 |
| 99 | 92269←93438 | 389 | *p45* | L | 103 | 36 |  | 104 | 59 |  | 90 | 62 | Orle90 |
| 100 | 93575→95875 | 766 | *p87/vp80* | ? | 104 | 12 |  | 105 | 16 |  | 91 | 21 | Orle91 |
| 101 | 95899→96066 | 55 | *ac110* | L | 110 | 38 |  | 106 | 43 |  | 92 | 46 | Agse67 |
| 102 | 96132→97211 | 359 | *odv-ec43* | L | 109 | 49 |  | 107 | 62 |  | 93 | 68 | Orle93 |
| 103 | 97267→97515 | 82 |  | L | 108 | 29 |  | 108 | 30 |  | 94 | 24 | Sf58 |
| 104 | 97535←98377 | 280 | *p13* | E,L | － | － |  | － | － |  | － | － | Se58 |
| 105 | 98497→99591 | 364 |  | ? | 112/113 | 28/21 |  | 109 | 23 |  | 96 | 27 | Orle96 |
| 106 | 99707←100807 | 366 | *p43* | E | 39 | 15 |  | － | － |  | － | － | Mavi28 |
| 107 | 100965←101684 | 239 | *ac106* | L | 106 | 57 |  | 140 | 62 |  | 99 | 67 | Tn102 |
| 108 | 101734←103209 | 491 |  | L | － | － |  | 141 | 16 |  | 101 | 17 | Hz103 |
| 109 | 103312←103806 | 164 |  | E | － | － |  | － | － |  | － | － | HearG4 99 |
| 110 | 103902←104519 | 205 | *pif-3* | E,L | 115 | 38 |  | 143 | 38 |  | 102 | 47 | Orle102 |
| 111 | 104643←105281 | 212 |  | E | － | － |  | 144 | 17 |  | 103 | 16 | MacoA67 |
| 112 | 105442→105906 | 154 | *sod* | E,L | 31 | 76 |  | 145 | 67 |  | 105 | 72 | MacoB65 |
| 113 | 107787→108143 | 118 |  | ? | － | － |  | － | － |  | － | － | Agse52 |
| 114 | 108173→108448 | 91 |  | E | 117 | 26 |  | － | － |  | 108 | 35 | Agse51 |
| 115 | 108600←109619 | 339 | *bro-b* | E |  |  |  |  |  |  |  |  | Hycu88 |
| 116 | 109785→110453 | 222 | *orf603* | E | 7 | 16 |  | － | － |  | － | － | Ac7 |
| 117 | 110580←111719 | 379 | *pif-2* | L | 22 | 65 |  | 119 | 64 |  | 112 | 70 | Orle112 |
| 118 | 111826←112380 | 184 | *pkip* | L | 24 | 20 |  | 110 | 31 |  | 113 | 31 | Tn139 |
| 119 | 112539←113201 | 220 | *lef-2* | E | 6 | 36 |  | 137 | 45 |  | 114 | 48 | Orer lef-2 |
| 120 | 113131←113496 | 121 |  | L | － | － |  | － | － |  | 115 | 13 | Hear117a |
| 121 | 113598→114395 | 265 | *p24* | L | 129 | 38 |  | － | － |  | 116 | 48 | Tn126 |
| 122 | 114415←114843 | 142 |  | L | － | － |  | 135 | 17 |  | 117 | 22 | Orle117 |
| 123 | 114963→115268 | 101 | *gp16* | E,L | 130 | 26 |  | － | － |  | 118 | 42 | Tn125 |
| 124 | 115394→116410 | 338 | *calyx/pep* | E,L | 131 | 25 |  | 136 | 42 |  | 109 | 44 | Chch121 |
| 125 | 116513←116890 | 125 |  | E,L | 19 | 28 |  | 159 | 31 |  | 122 | 31 | Sf40 |
| 126 | 116885→118096 | 403 |  | L | 18 | 13 |  | 158 | 13 |  | 123 | 22 | MacoA56 |
| 127 | 118192→119409 | 405 | *alk-exo* | L | 133 | 34 |  | 157 | 38 |  | 124 | 42 | MacoB47 |
| 128 | 119890→121119 | 409 | *fgf* | L | 32 | 24 |  | 156 | 21 |  | 125 | 19 | Agse39 |
| 129 | 121181←121423 | 80 | ***Clbi129*** | L | － | － |  | － | － |  | － | － | － |
| 130 | 121480←123096 | 538 | *pif-1* | L | 119 | 44 |  | 155 | 36 |  | 126 | 51 | Orle126 |
| 131 | 123210←123986 | 258 | *bro-c* | ? |  |  |  |  |  |  |  |  | Lese153 |
| 132 | 124156←125334 | 392 | *38.7K* | ? | 13 | 21 |  | 122 | 23 |  | 133 | 25 | MacoB31 |
| 133 | 125398←126090 | 230 | *lef-1* | E | 14 | 35 |  | 123 | 42 |  | 132 | 49 | Orle132 |
| 134 | 126159→126515 | 118 |  | E,L | － | － |  | 124 | 26 |  | 131 | 29 | Orle131 |
| 135 | 126646→128244 | 532 | *egt* | E | 15 | 39 |  | 125 | 43 |  | 130 | 53 | Tn133 |
| 136 | 128343→128972 | 209 |  | E | － | － |  | 127 | 20 |  | 129 | 28 | Tn135 |
| 137 | 129026→129757 | 243 |  | E,L | 17 | 17 |  | 128 | 21 |  | 46 | 12 | Ecob120 |
| 138 | 129833←132943 | 1036 |  | E,L | － | － |  | 129 | 31 |  | 128 | 19 | Ld129 |
| 139 | 133108→135216 | 702 | *F protein* | E,L | 23 | 11 |  | 130 | 56 |  | 127 | 49 | Ld130 |

* The left and right boundaries are given by nucleotide numbers.

† The orientation of a gene is indicated as ‘→’ or ‘←’ indicating the same or reverse orientation as the polyhedrin gene, respectively.

‡ Baculovirus core genes are double underlined, additional genes common to Lepidopteran NPVs are underlined, genes unique to ClbiNPV are shown in bold.

§ Presence of predicted early (E) (CAGT) or late (L) (TAAG) promoter motifs 150 nt upstream of the ATG. The question mark means that no conserved baculovirus E or L promoter motif is present.
