# Supplementary material for: Genomic sequence, organization and characteristics of a new nucleopolyhedrovirus isolated from Clanis bilineata larva
Source: BMC Genomics. 2009 Feb 25;10:91. doi: 10.1186/1471-2164-10-91 (PMC2650706; doi:10.1186/1471-2164-10-91)
Supplement: Additional file 3 — Overlapping ORF pairs in the sequenced Alphabaculovirus genomes. Overlapping ORFs in ClbiNPV were shown and the overlapping ORF pairs in all sequenced Alphabaculovirus genomes were analysed. [file 1471-2164-10-91-S3.doc]

## Additional file 3 Overlapping ORF pairs in the sequenced Alphabaculovirus genomes.

1. Overlapping ORF pairs in ClbiNPV genome.

| Overlapping ORF pairs of ClbiNPV | Homologous ORF of AcMNPV | Size of the overlap (bp) |
| --- | --- | --- |
| *Clbi1*/*Clbi2 (polyhedrin*/*orf1629)* | *ac8*/*ac9* | 4 |
| *Clbi4*/*Clbi5 (hoar*/*Clbi5)* | －/－ | 77 |
| *Clbi12*/*Clbi13(odv-e18*/*p49)* | *ac143*/*ac142* | 56 |
| *Clbi17*/*Clbi18(p74*/*Clbi18)* | *ac138*/－ | 44 |
| *Clbi28*/*Clbi29 (39K*/*lef-11)* | *ac36*/*ac37* | 65 |
| *Clbi29*/*Clbi30 (lef-11*/*ac38)* | *ac37*/*ac38* | 76 |
| *Clbi34*/*Clbi35 (ac52*/*Clbi35)* | *ac52*/－ | 4 |
| *Clbi43*/*Clbi44 (Chch42*/*Agse115)* | －/－ | 4 |
| *Clbi44*/*Clbi45 (Agse115*/*lef-10)* | －/*ac53a* | 53 |
| *Clbi45*/*Clbi46 (lef-10*/*vp1054)* | *ac53a*/*ac54* | 137 |
| *Clbi48*/*Clbi49 (ac55*/*Clbi49)* | *ac55*/－ | 40 |
| *Clbi50*/*Clbi51 (ac57*/*ac59)* | *ac57*/*ac59* | 23 |
| *Clbi56*/*Clbi57 (Clbi56*/*Clbi57)* | －/－ | 4 |
| *Clbi70*/*Clbi71 (Clbi70*/*iap-2)* | －/*ac71* | 67 |
| *Clbi73*/*Clbi74 (ac68*/*lef-3)* | *ac68*/*ac67* | 137 |
| *Clbi75*/*Clbi76 (Clbi75*/*desmoplakin)* | －/*ac66* | 59 |
| *Clbi80*/*Clbi81 (vlf-1*/*ac78)* | *ac77*/*ac78* | 8 |
| *Clbi83*/*Clbi84 (ac81*/*tlp20)* | *ac81*/*ac82* | 368 |
| *Clbi84*/*Clbi85 (tlp20*/*vp91)* | *ac82*/*ac83* | 149 |
| *Clbi89*/*Clbi90 (ac92*/*ac93)* | *ac92*/*ac93* | 2 |
| *Clbi92*/*Clbi93 (helicase*/*ac96)* | *ac95*/*ac96* | 32 |
| *Clbi94*/*Clbi95 (38K*/*lef-5)* | *ac98*/*ac99* | 27 |
| *Clbi95*/*Clbi96 (lef-5*/*p6.9)* | *ac99*/*ac100* | 7 |
| *Clbi98*/*Clbi99 (p12*/*p45)* | *ac102*/*ac103* | 8 |
| *Clbi119*/*Clbi120 (lef-2*/*Hear117a)* | *ac6*/－ | 71 |
| *Clbi125*/*Clbi126 (ac19*/*ac18)* | *ac19*/*ac18* | 6 |

1. Overlapping ORF pairs in Group I Alphabaculovirus.

| Group I Alphabaculovirus | No. overlapping ORFs | No. ORFs | AcMNPV ORF | | | | | | | | |
| --- | --- | --- | --- | --- | --- | --- | --- | --- | --- | --- | --- |
|  |  |  | 43/44 | 68/69 | 73/74 | 80/81 | 81/82 | 82/83 | 95/96 | 98/99 | 102/103 |
| BmNPV (T3) | 21 | 143 | + | + | + | + | + | + | + | + | + |
| AcMNPV (C6) | 23 | 156 | + | + | + | + | + | + | + | + | + |
| RoMNPV | 29 | 149 | + | + | + | + | + | + | + | + | + |
| MaviMNPV | 29 | 126 | + | + | + | + | + | + | + | + | + |
| PlxyMNPV (CL1) | 30 | 152 | + | + | + | + | + | + | + | + | + |
| CfMNPV | 32 | 146 | + | + | + | + | + | + | + | + | + |
| CfDefNPV | 33 | 149 | + | + | + | + | + | + | + | + | + |
| EppoNPV | 33 | 136 | + | + | + | + | + | + | + | + | + |
| OpMNPV | 34 | 152 | + | － | － | + | + | + | + | + | + |
| HycuNPV | 35 | 148 | － | + | + | + | + | + | + | + | + |
| AnpeNPV | 40 | 147 | + | + | + | + | + | + | + | + | + |
| AgMNPV (D2) | 42 | 152 | + | + | + | + | + | + | + | + | + |

1. Overlapping ORF pairs in Group II Alphabaculovirus.

| Group II Alphabaculovirus | No. overlapping ORFs | No. ORFs | AcMNPV ORF | | | | | | | | | |
| --- | --- | --- | --- | --- | --- | --- | --- | --- | --- | --- | --- | --- |
|  |  |  | 53a/54 | 57/59 | 67/68 | 80/81 | 81/82 | 82/83 | 89/90 | 95/96 | 98/99 | 102/103 |
| EcobNPV (A1) | 18 | 126 | + | + | + | － | + | + | － | + | + | + |
| OrleNPV (CSF-77) | 18 | 135 | － | + | + | － | + | + | + | + | + | + |
| HearSNPV (C1) | 22 | 137 | + | － | － | + | + | + | + | + | + | + |
| HearSNPV (G4) | 23 | 135 | + | － | － | + | + | + | + | + | + | + |
| HearSNPV (NNg1) | 24 | 143 | + | － | － | + | + | + | + | + | + | + |
| HzSNPV | 24 | 139 | + | － | － | + | + | + | + | + | + | + |
| ChchNPV | 24 | 151 | + | + | + | － | + | － | + | + | + | + |
| AgseNPV | 24 | 153 | + | + | + | － | + | － | + | + | + | + |
| AdhoNPV (ADN001) | 25 | 125 | + | + | + | + | + | + | + | + | + | + |
| ClbiNPV (DZ1) | 26 | 139 | + | + | + | － | + | + | － | + | + | + |
| SeMNPV | 28 | 139 | + | + | + | + | + | + | + | + | + | + |
| TnSNPV | 29 | 145 | + | + | + | + | + | + | + | + | + | + |
| SpltMNPV(G2) | 31 | 141 | + | － | － | + | + | + | － | + | + | + |
| LdMNPV | 33 | 164 | + | + | + | + | + | + | + | + | + | + |
| LeseNPV(AH1) | 34 | 169 | + | + | + | + | + | + | + | + | + | + |
| SpltNPV II | 34 | 147 | + | + | + | + | + | + | － | + | + | + |
| AdorNPV | 36 | 121 | + | － | + | + | + | + | + | + | + | + |
| MacoNPV(A) | 36 | 169 | + | + | + | － | + | + | + | + | + | + |
| HearMNPV | 39 | 162 | + | + | + | + | + | + | + | + | + | + |
| MacoNPV(B) | 40 | 168 | + | + | + | + | + | + | + | + | + | + |
| SfMNPV(3AP2) | 41 | 142 | + | + | + | + | + | + | + | + | + | + |
| AgipMNPV | 42 | 163 | + | + | + | + | + | + | + | + | + | + |
